# Supplementary material for: Multimodal predictions of end stage chronic kidney disease from asymptomatic individuals for discovery of genomic biomarkers
Source: BMC Nephrol. 2026 May 12;27:405. doi: 10.1186/s12882-026-05016-7 (PMC13339857; doi:10.1186/s12882-026-05016-7)
Supplement: Supplementary file 1 — Supplementary Material 1 [file 12882_2026_5016_MOESM1_ESM.pdf]

Supplementary Tables

Table S1: Subject Characteristics

(a) Counts and frequencies

Quantity	Counts	Frequencies (%)
Sex (male)	64637	47.27
Type 2 Diabetes (T2D)	15332	11.21
preT2D	11625	8.50
Hyper Tension (HT)	53620	39.21
preHT	43720	31.97
Congestive Heart Failure (CHF)	6444	4.71
preCHF	3702	2.71
CKD	80853	59.13
Threshold age of 60 (t_age)	122479	89.56
Threshold age 60 at CKD diagnosis (t_CKD_age)	49345	36.08
End Stage Renal Disease (ESRD)	371	0.27
All CKD stages with MRI data	2151	1.57
CKD stages 0,1,2 with MRI data	188	0.13
ESRD with MRI data	210	0.15
BlackBrit	1629	1.19
SouthAsian	2688	1.96
Has Genetics	130645	95.54

(b) Averages

Quantity	Averages
Age	71.1
CKD diagnosis age	61.3

(a) CKD vs. phenotypes Logistic Regression

Feature	OR	95CI-	95CI+	P-val
preHT	2.023	1.972	2.076	0.000E+00
preT2D	2.112	2.012	2.218	2.642E-198
preCHF	1.822	1.675	1.981	1.026E-44
t_age	1.530	1.476	1.586	8.506E-119
sex	0.852	0.833	0.871	8.306E-45
BlackBrit	2.048	1.822	2.301	2.038E-33
SouthAsian	2.837	2.570	3.132	7.974E-95

(b) ESRD vs. phenotypes Logistic Regression

Feature	OR	95CI-	95CI+	P-val
preHT	2.273	1.800	2.870	5.134E-12
preT2D	2.090	1.640	2.665	2.685E-09
preCHF	1.706	1.209	2.405	2.337E-03
t_age	0.734	0.455	1.184	2.048E-01
t_CKD_age	2.946	2.304	3.767	7.014E-18
sex	2.348	1.871	2.945	1.597E-13
BlackBrit	2.894	1.677	4.991	1.337E-04

Table S2: ESRD vs. Phenotypes: Logistic Regression

Table S3: Cox regression phenotypic coefficients predicting time to ESRD

covariate	exp(coef)	exp(coef) lower 95%	exp(coef) upper 95%	p
preHT	0.720	0.444	1.169	0.184
preT2D	1.508	0.937	2.429	0.091
preCHF	1.760	0.691	4.480	0.236
Thiazide	1.134	0.587	2.188	0.708
s_age	0.596	0.408	0.873	0.008
t_CKD_age	2.301	1.159	4.570	0.017
sex	1.072	0.657	1.748	0.782
BlackBrit	0.555	0.103	2.975	0.492

Table S4: GO Enrichment

Type	**Count**	DE	xDE	DxE	xDxE	Pval
Glomerulus	25	19	6007	18830	2300270	1.74E-06
Nephron	40	14	6237	18835	2300040	1.96E-09
Metanephric	56	18	4254	18831	2302023	0.002693
Kidney	142	85	19964	18764	2286313	2.49E-11
Renal	132	169	14304	18680	2291973	6.07E-06
Kidney/Renal	253	245	32557	18604	2273720	0.203546

(a) CKD vs. the CKD genotype and phenotypes Logistic Regression

	OR	95CI-	95CI+	P-val
preHT	2.013	1.961	2.067	0.000E+00
preT2D	2.095	1.992	2.203	6.848E-183
preCHF	1.805	1.657	1.966	8.387E-42
sex	0.854	0.835	0.873	4.628E-42
t_age	1.561	1.505	1.621	2.593E-122
BlackBrit	1.395	1.092	1.783	7.786E-03
SouthAsian	2.712	2.439	3.017	1.847E-75
CKDgeno	0.898	0.874	0.922	1.001E-15

(b) ESRD vs. CKD genotypes and phenotypes Logistic Regression

	OR	95CI-	95CI+	P-val
preHT	2.290	1.801	2.912	1.403E-11
preT2D	1.978	1.531	2.556	1.833E-07
preCHF	1.653	1.148	2.380	6.914E-03
t_age	0.502	0.292	0.863	1.261E-02
t_CKD_age	4.313	3.165	5.878	2.146E-20
CKDgeno	1.239	0.975	1.575	7.921E-02
sex	2.423	1.910	3.073	2.990E-13
BlackBrit	1.456	0.202	10.491	7.090E-01
SouthAsian	1.221	0.644	2.314	5.412E-01

Table S5: ESRD vs. CKD Genotypes and Phenotypes: Logistic Regression

Table S6: Cox regression phenotypic/genotypic coefficients predicting time to ESRD

covariate	exp(coef)	exp(coef) lower 95%	exp(coef) upper 95%	p
preHT	0.857	0.500	1.469	0.576
preT2D	1.368	0.842	2.221	0.206
preCHF	1.110	0.340	3.621	0.862
Thiazide	0.996	0.509	1.949	0.990
s_age	0.671	0.427	1.055	0.084
t_CKD_age	2.054	0.811	5.204	0.129
sex	0.840	0.500	1.411	0.510
CKDgeno	1.510	0.896	2.546	0.122

Table S7: GO-term SNP Counts ≥ 10 of Benjamini-Hochberg SNPs significant at 1×10^{-4}

GO-term	Count
Protein binding	104
Nucleoplasm	61
Acid-thiol ligase activity	48
Fatty acid metabolic process [57]	48
Malonyl-CoA synthetase activity [58]	48
Long-chain fatty-acyl-CoA biosynthetic process [57]	48
Fatty acid biosynthetic process [57]	48
ATP binding	48
Malonate catabolic process	48
Mitochondrial matrix	48
Mitochondrion	48
Very long-chain fatty acid-CoA ligase activity [57]	48
Plasma membrane	44
Metal ion binding	29
Extracellular matrix organization [59]	29
G protein-coupled receptor activity	23
Lung connective tissue development	22
Parturition	22
Myofibroblast differentiation	22
Nipple morphogenesis	22
Hormone binding	22
Hormone-mediated signaling pathway	22
Cytosol	21
Membrane	19
miRNA-mediated gene silencing	10
Rndoplasmic reticulum membrane	10
Positive regulation of nuclear-transcribed mRNA poly(A) tail shortening	10
Extracellular region	10
P-body	10
RNA binding	10
miRNA-mediated gene silencing by inhibition of translation	10
Regulation of nuclear-transcribed mRNA catabolic process, deadenylation-dependent decay	10

Table S8: Gene SNP Counts of Benjamini-Hochberg significant SNPs at $p \leq 1 \times 10^{-4}$

Gene	Count
ACSF3	48
RXFP1 [60]	22
TNRC6C	10
LPCAT1 [61]	9
COL4A1 [62]	7
KCNQ2	4
LIMA1	4
TMOD1 [63]	2
SPACA7	2
KLF3 [64]	2
TNNT3	1
ADGRA3	1
DLG2	1
AKR1C1	1
GLI3	1
AFM	1
GRID2IP	1
NUP205 [65]	1
KCNH5	1
PLGRKT	1
TCP11	1
SLC16A11	1

(a) CKD vs. PCA and phenotypes and CKD geno Logistic Regression

	OR	95CI-	95CI+	P-val
preHT	2.077	2.024	2.132	0.000E+00
preT2D	2.057	1.957	2.163	6.423E-175
preCHF	1.821	1.672	1.984	5.075E-43
PC0s	1.163	1.147	1.179	5.227E-103
PC1s	0.969	0.958	0.981	6.046E-07
PC2s	0.983	0.971	0.995	5.782E-03
CKDgeno	0.913	0.889	0.937	1.306E-11

(b) CKD geno vs. PCA and phenotypes Logistic Regression

	OR	95CI-	95CI+	P-val
preHT	0.996	0.968	1.024	7.652E-01
preT2D	1.000	0.952	1.051	9.882E-01
preCHF	1.004	0.927	1.088	9.163E-01
PC0s	0.602	0.580	0.626	2.832E-147
PC1s	1.105	1.080	1.131	1.964E-17
PC2s	0.969	0.946	0.994	1.338E-02

Table S9: PCA impacts on CKD and relation to CKD geno

Table S10: List of SNPs associated with CKD, Energy, Non-uniformity and CVD (see attached file). **ID** is the SNP identification number, **OR** is Odds Ratio, **L95** is the value for the lower bound 5% Confidence interval, **U95** is the value for the upper bound 5% Confidence interval, **P** is the raw P-value, **Pbh** is the Bonferroni corrected P-value, **symbol** is the symbol of the gene associated with the SNP, i.e in the 10kb region.

Table S11: Radiomics image processing table

Parameter	Value
normalizeScale	100
binWidth	5
preCrop	True

Table S12: Deep learning net hyper-parameters

Parameter	Value
Learning arguments	
learning rate	1e-4
weight decay	1e-5
batch size	4
Resnet arguments	
first channel dim	32
first stride	2
stem kernel size	[3, 3, 3]
stem stride	[2, 2, 2]
layers	[2, 2, 2, 2]
Multilayer Perceptron arguments	
dropout rate	0
encoder dropout rate	0
mlp hidden layers	2
mlp hidden dim	128
head hidden layers	1
head bias	True
use batch normalization	True
Vision Transformer (ViT) arguments	
patch size	[16, 16, 16]
token dim	512
dim head	64
mlp dim	256
depth	8
heads	8
dropout	0
emb dropout	0
num cls tokens	1

Supplementary Figures

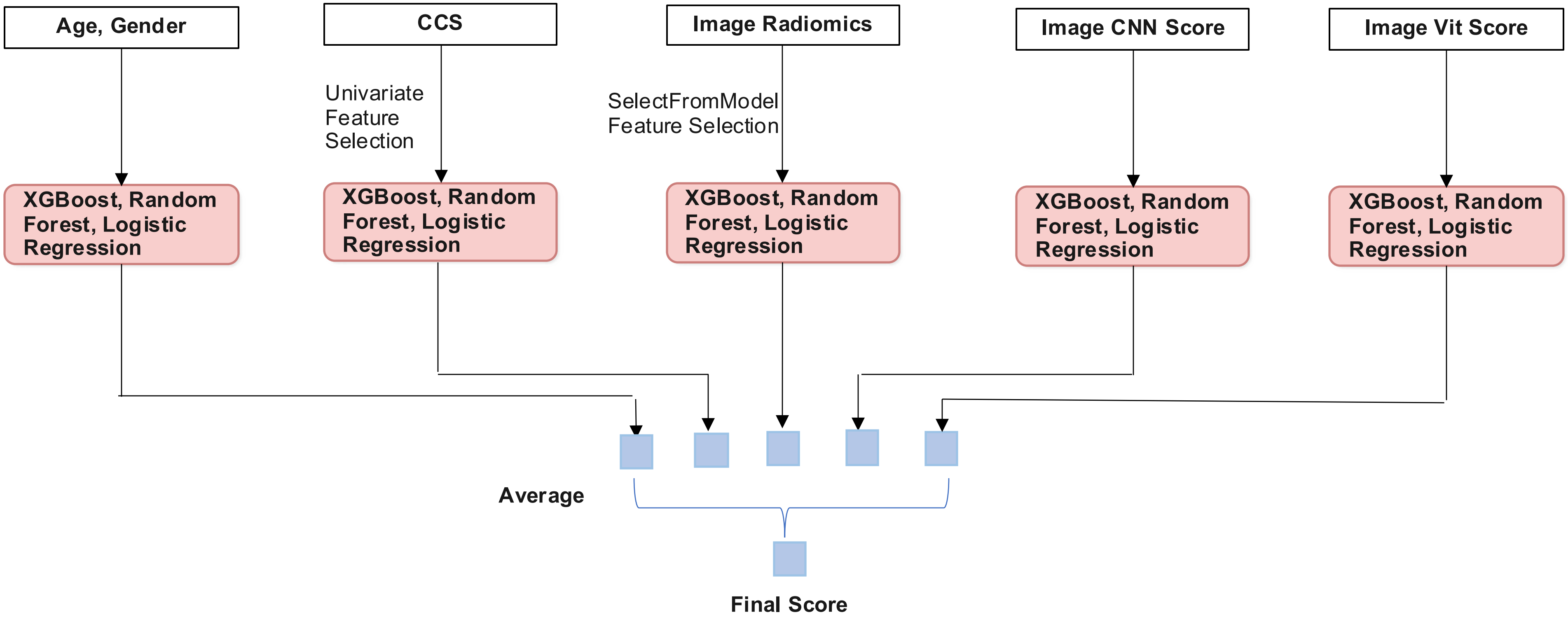

Figure S1. General approach for modality integration.
For each of 3 data modalities and 5 models (Demographic, Clinical Codes, Image Radiomics/CNN/Vit) features were selected and implemented in a XGBoost, Random Forest, Logistic Regression classifier to predict 5-year outcome for ESRD. A voting scheme was used to determine the final prediction.

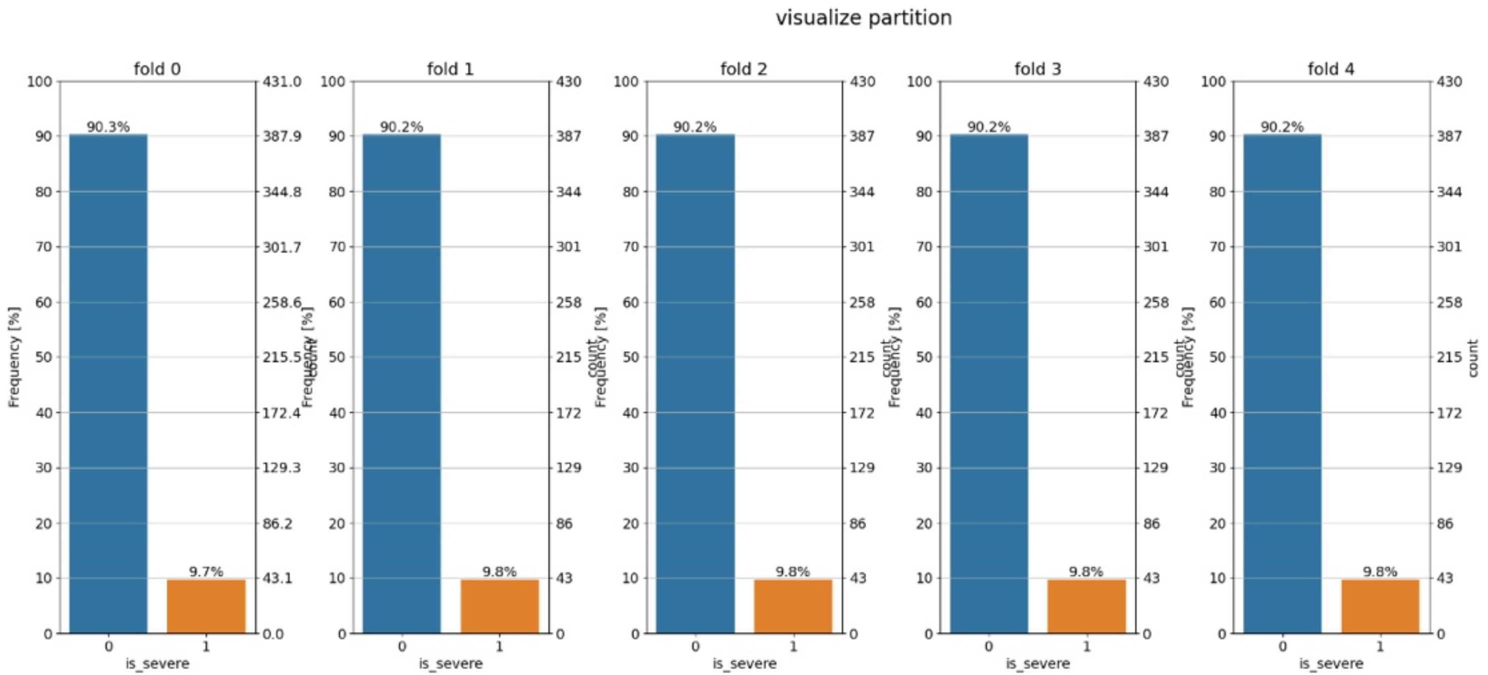

Figure S2. Data Splits for each data modality. Blue bars represent percentage of patients with early CKD and orange bars with ESRD for each cross-validation fold.

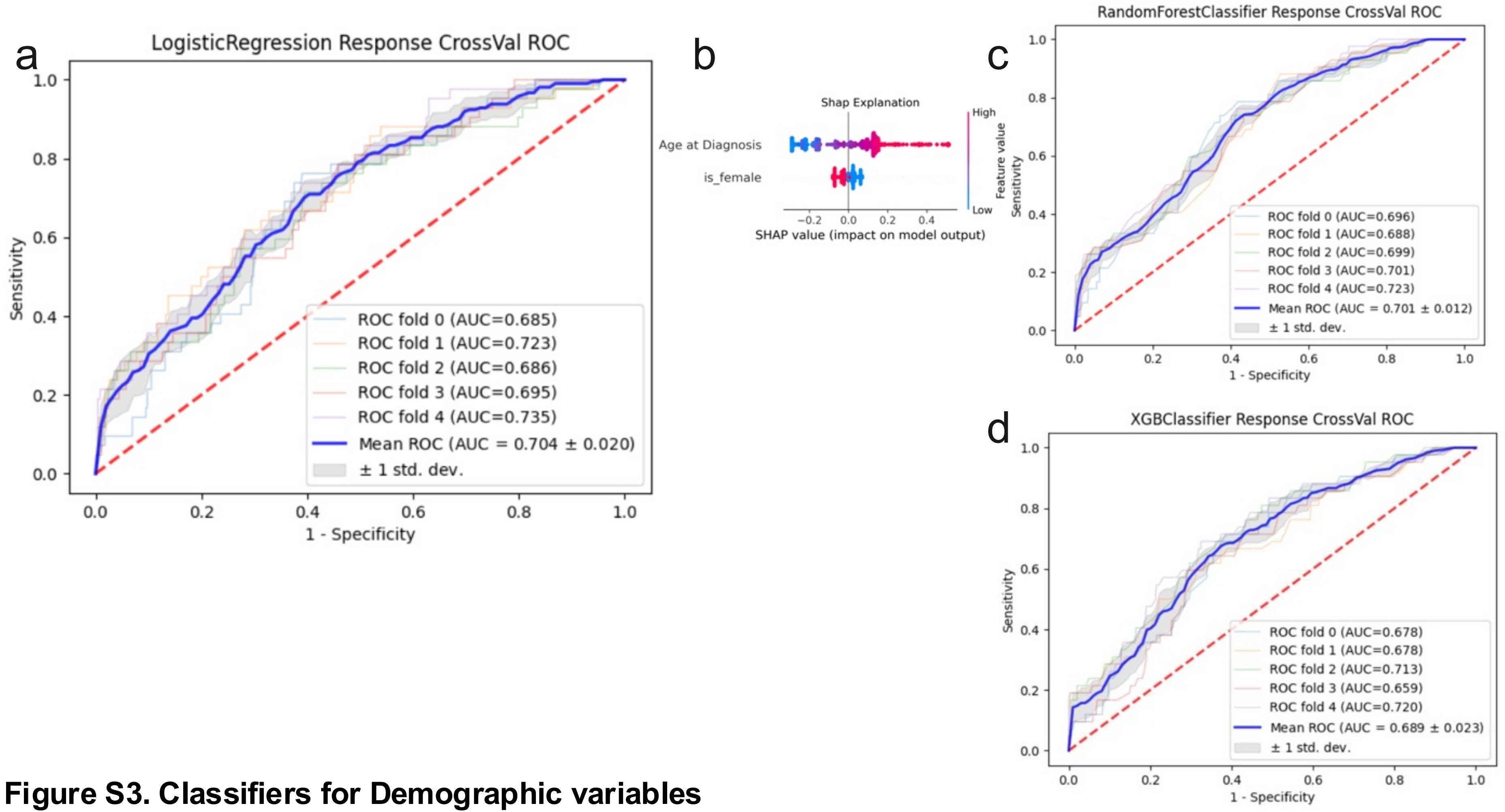

Figure S3. Classifiers for Demographic variables

a. Logistic Regression **b.** SHAP values **c.** Random Forest **d.** XGBoost classifier

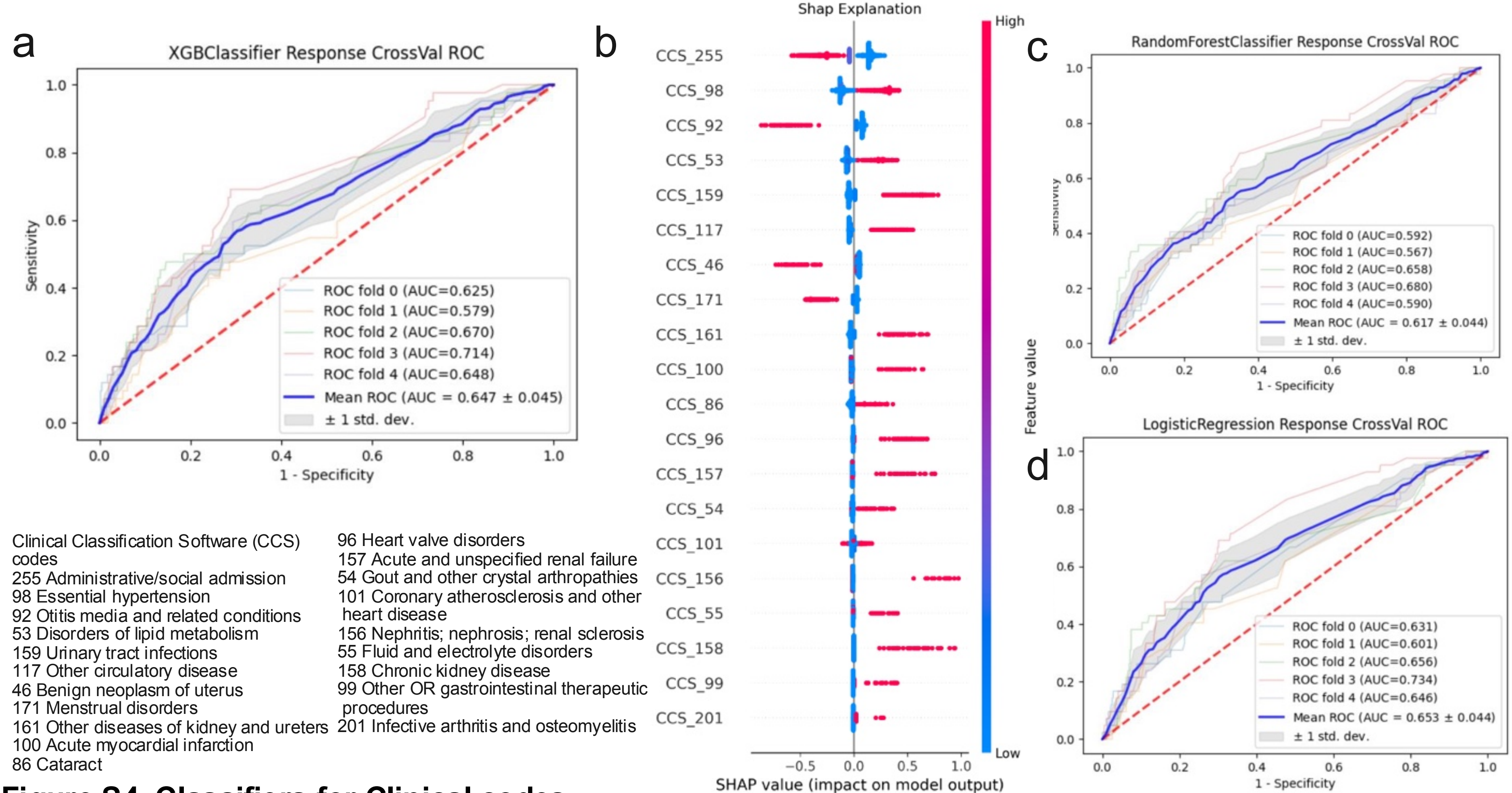

Figure S4. Classifiers for Clinical codes

a. XGBoost classifier **b.** SHAP values **c.** Random Forest **d.** Logistic Regression

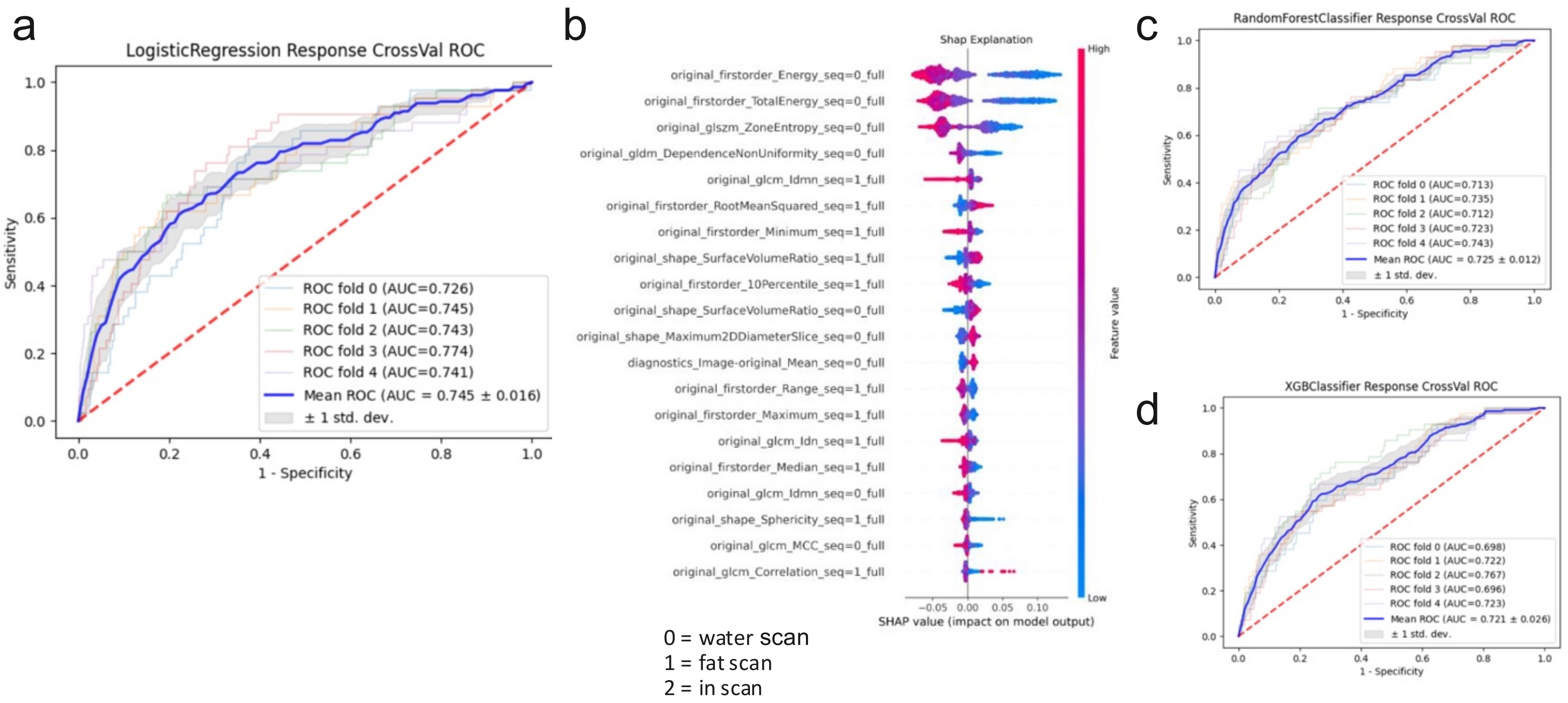

Figure S5. Classifiers for Image Radiomics
a. Logistic Regression **b.** SHAP values **c.** Random Forest **d.** XGBoost classifier

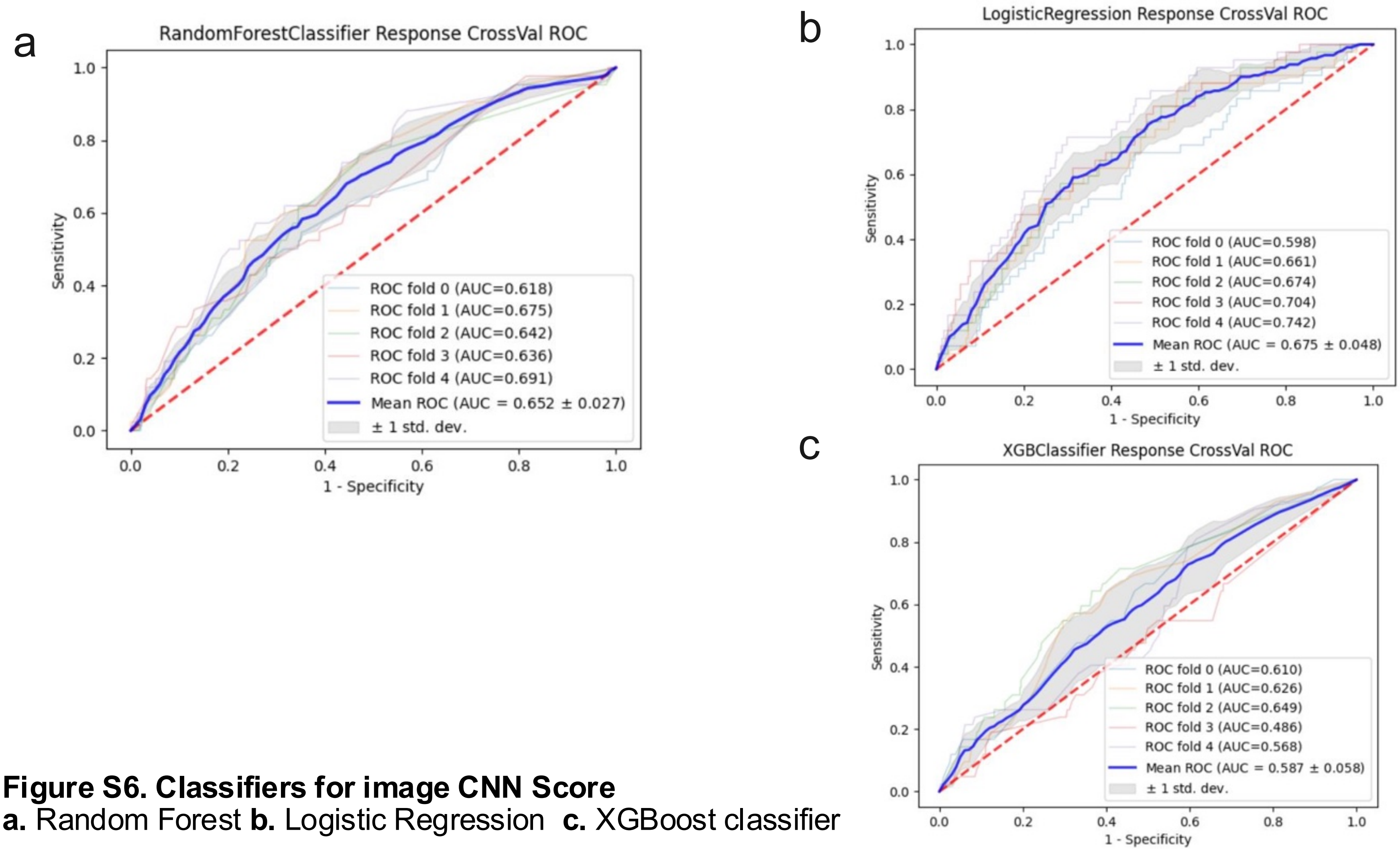

Figure S6. Classifiers for image CNN Score

a. Random Forest **b.** Logistic Regression **c.** XGBoost classifier

a

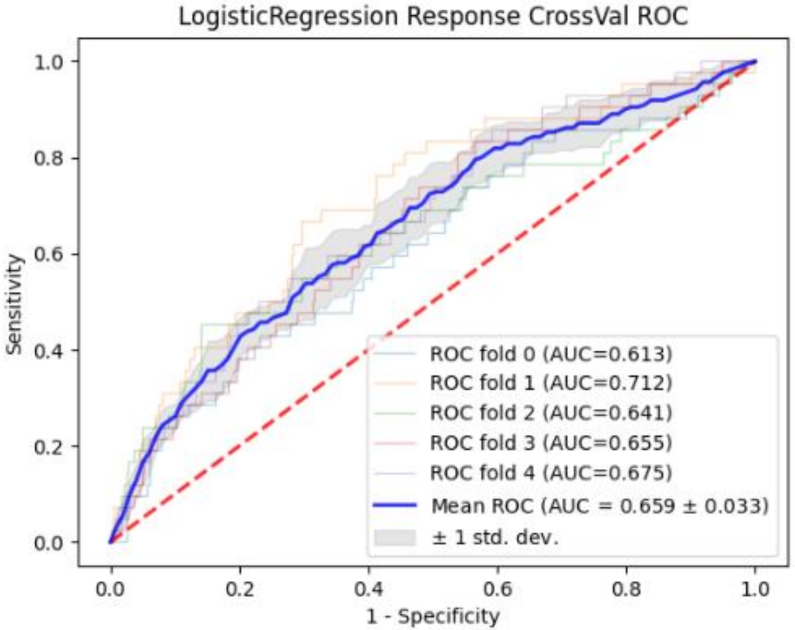

b

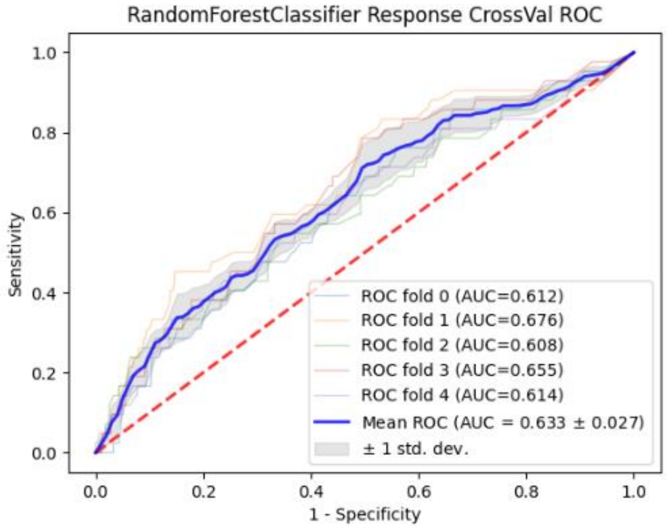

c

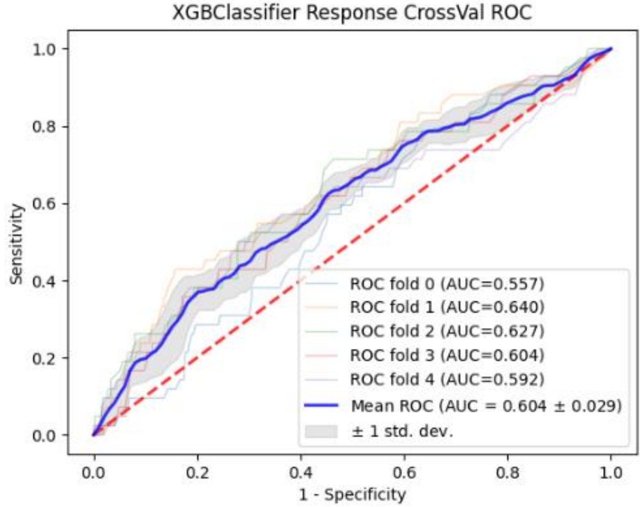

Figure S7. Classifiers for image ViT Score (14/5 last epoch embeddings were used as features) **a.** Logistic Regression **b.** Random Forest **c.** XGBoost classifier

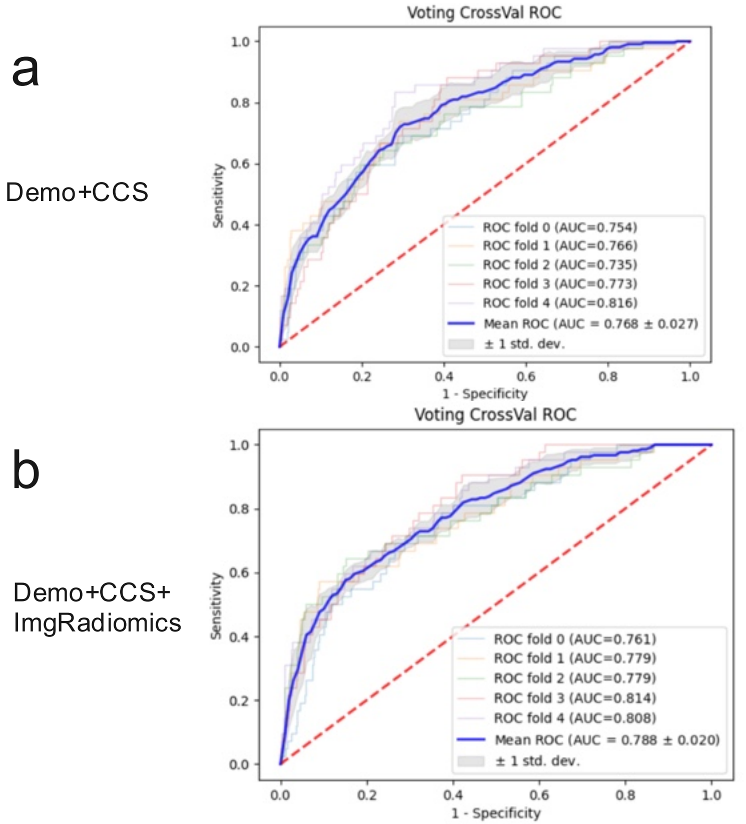

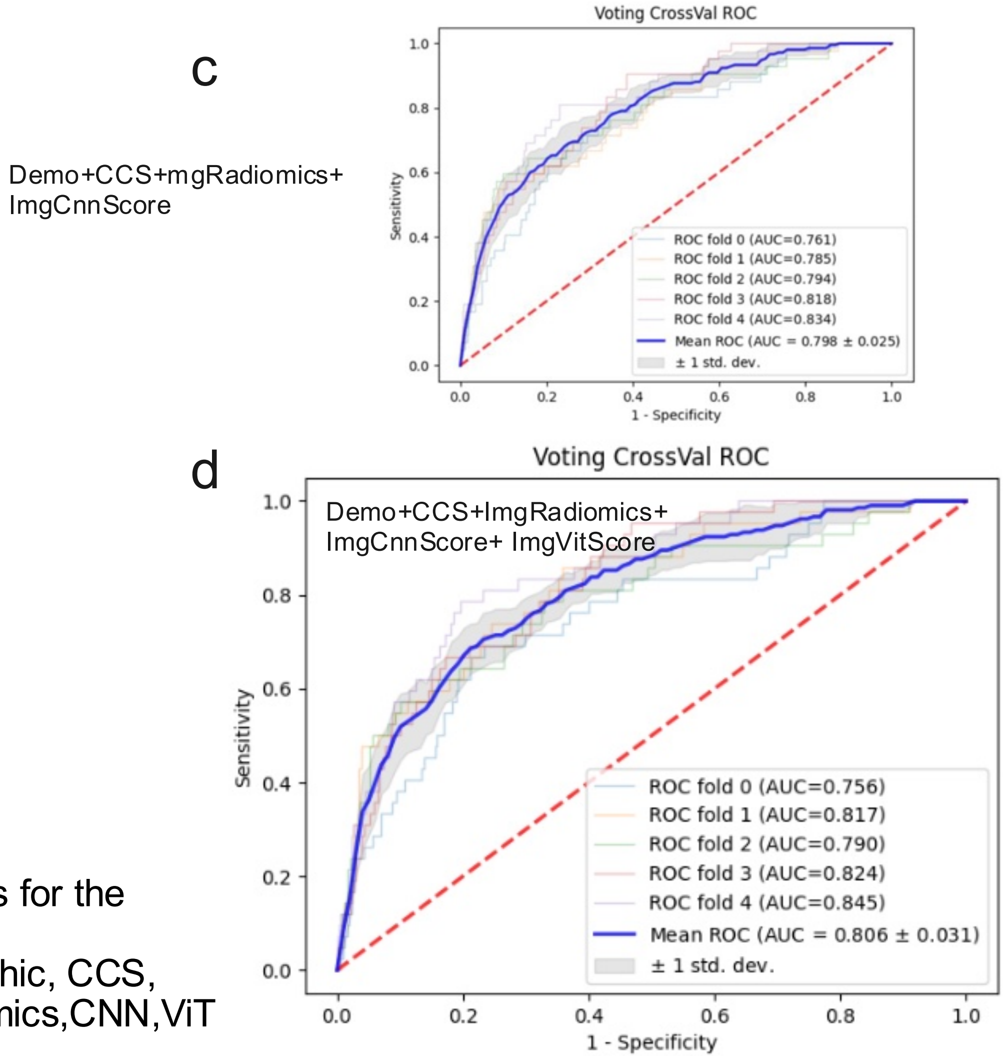

Figure S8. Ensemble models Show are the results for the following combinations **a.** Demographic and CCS **b.** Demographic, CCS and Radiomics **c.** Demographic, CCS, Radiomics and CNN **d.** Demographic, CCS, Radiomics,CNN,ViT

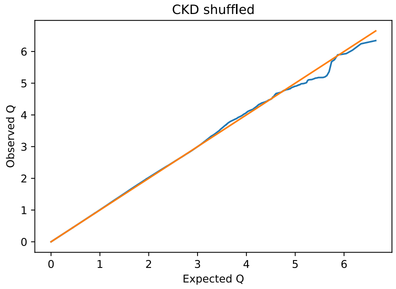

(a) Shuffled clinical variables; expansion factor $lambda = 0.999$.

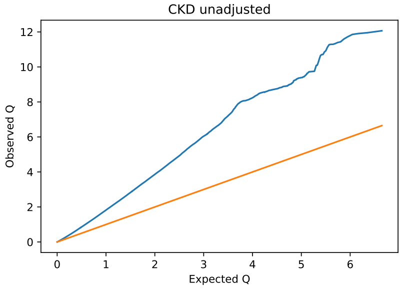

(b) CKD with no population adjustment; expansion factor $lambda = 2.157$.

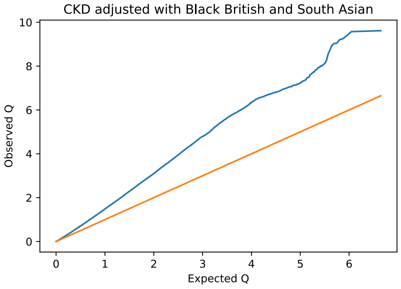

(c) CKD with population adjustment; expansion factor $lambda = 1.663$.

Fig. S9: QQ plots exploring population stratification baselines, selected features, and populations

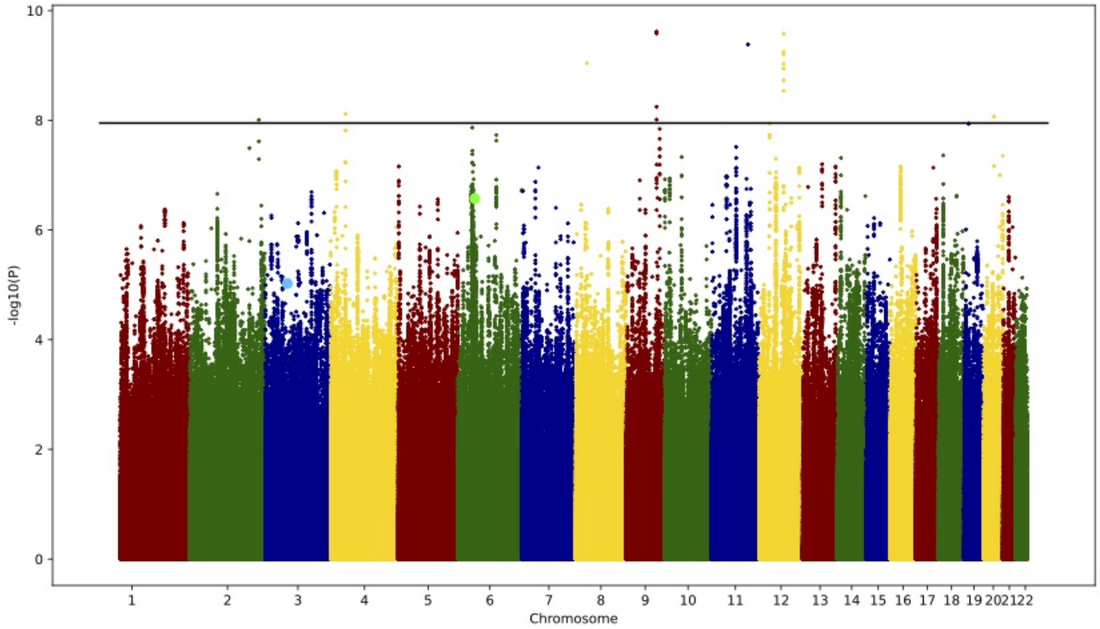

Fig. S10: Manhattan plot for CKD logistic regression including population adjustments. The x axis is the chromosomal location of SNP and the y axis the strength of association -log10(P value). Variants rs1383063 and rs12191777 are represented by larger light blue and light green dots respectively. Line represents the limit of significance with Bonferroni correction.

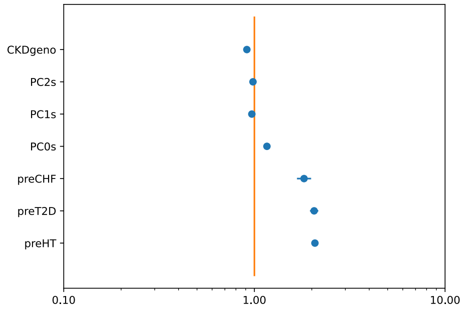

(a) PCA predicting CKD

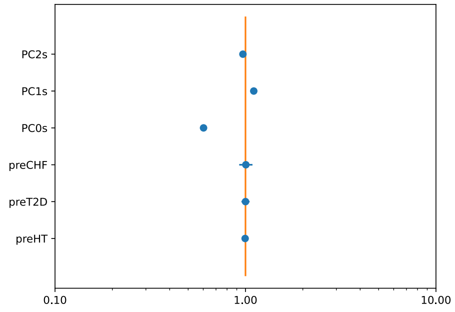

(b) PCA predicting CKD geno

Fig. S11: Logistic Regressions predicting CKD and CKD geno with leading principal components, pre CKD hypertension, Type II Diabetes, and Congestive Heart Failure.

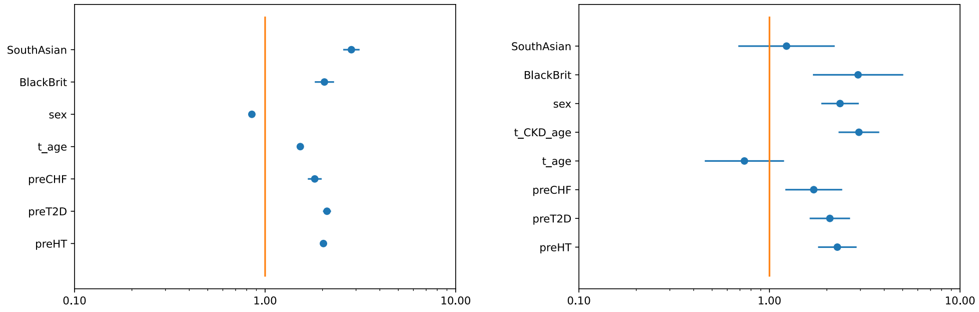

(a) CKD

(b) ESRD

Fig. S12: Logistic Regressions predicting CKD and ESRD with pre CKD hypertension, Type II Diabetes, Congestive Heart Failure, sex, age, and Black British status, South Asia status, and age of CKD diagnosis. t_age refers to binary-threshold age of 60 years.

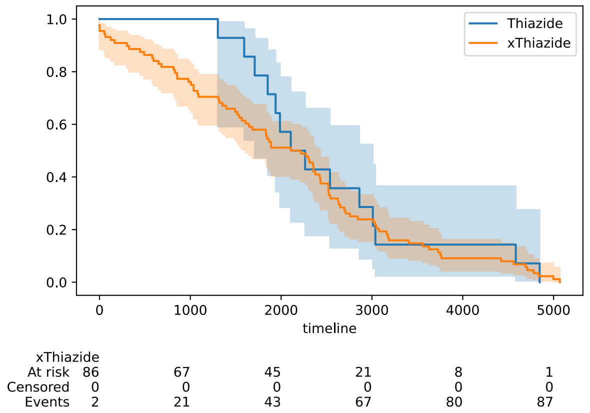

(a) Kaplan-Meier analysis

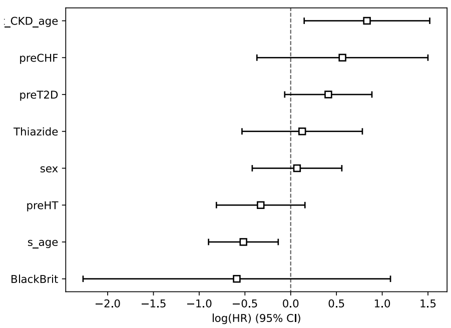

(b) Cox regression variables.

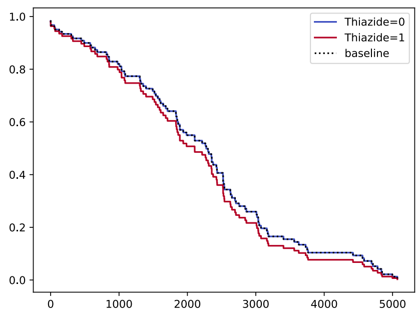

(c) Cox regression impact of thiazides.

Fig. S13: Hazard Ratio analysis of time to ESRD from CKD diagnosis. s_age refers to centered and standard error scaled age.

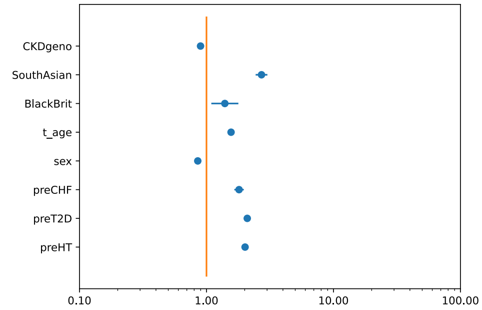

(a) CKD

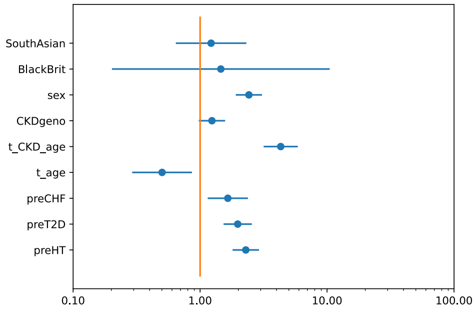

(b) ESRD

Fig. S14: Logistic Regressions predicting CKD and ESRD with CKD significant SNPs, pre CKD hypertension, Type II Diabetes, Congestive Heart Failure, sex, age, and Black British status, South Asia status, and age of CKD diagnosis.

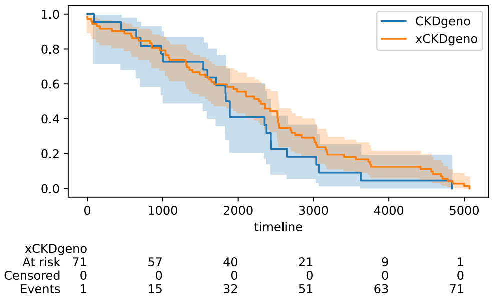

(a) Kaplan-Meier analysis

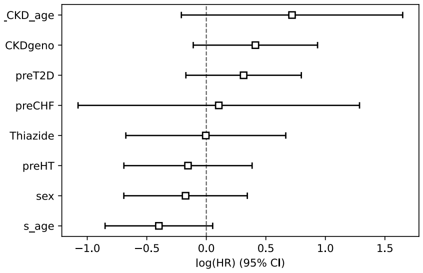

(b) Cox regression variables.

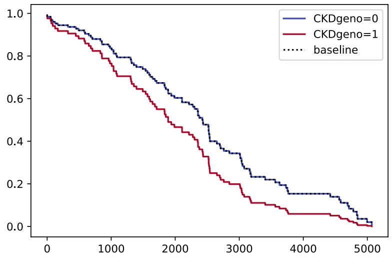

(c) Cox regression impact of CKDgeno.

Fig. S15: Hazard Ratio analysis of time to ESRD from CKD Genotypes. Censored or patients with ESRD happening $\geq$ 6000 days were excluded as also were patients were CKDGeno was not defined or missing GO-term derived kidney SNPs. 94 individuals were analyzed in total, 71 xCKDgeno and 23 CKDgeno.

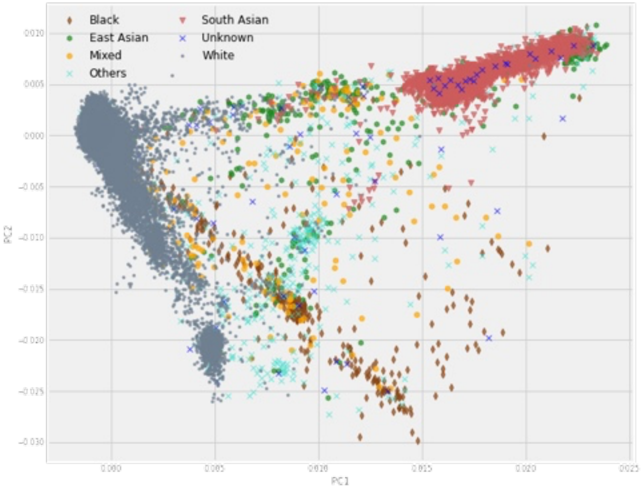

(a) PCA showing population relationships to stratification

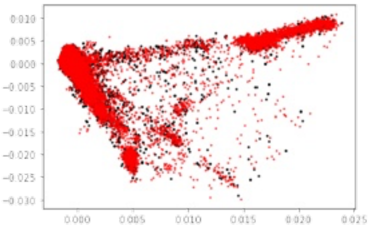

(b) PCA showing CKD (in red) relationship to stratification

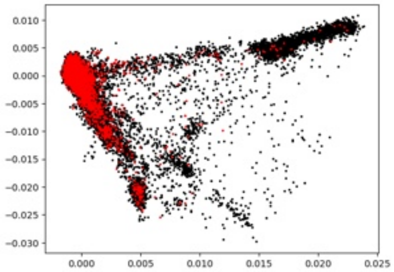

(c) PCA showing CKDgeno (in red) genetic relationship to stratification

Fig. S16: PCA showing relationships between CKD and CKDgeno to population stratification.

a

	SEX CODE (Male:1, Female:0)	Viral Infection (NCCS_1_3)	Disease of the heart NCCS7_2	# of patients	STD	# of uncensored patients	STD	cindex		test_t_mae		T2E	
				mean	std	mean	std	mean	std	mean	std	mean	std
Whole dataset	-	-	-	9954	0.447	31	0.447	0.634	0.036	42.77	1.07	94.74	0.18
	0	0	0	2105	42.980	3	1.342	0.616	0.068	38.722	9.87	90.517	1.059
	0	1	0	2837	10.237	6	1.673	0.626	0.094	32.465	10.1	97.159	0.750
	0	0	1	114	11.726	0	0.447	0.619		42.301		75.685	5.561
	0	1	1	366	14.957	2	1.517	0.701	0.173	57.271	13.7	95.276	2.468
	1	0	0	1852	48.484	6	2.588	0.633	0.147	45.025	21.9	93.383	1
	1	1	0	2083	40.833	11	2.828	0.586	0.075	42.971	5.44	98.672	1.264
	1	0	1	186	15.418	2	1.304	0.777	0.204	57.321	49.5	81.766	4.153
	1	1	1	408	18.322	2	1.517	0.575	0.084	42.551	8.40	96.513	2.190

b

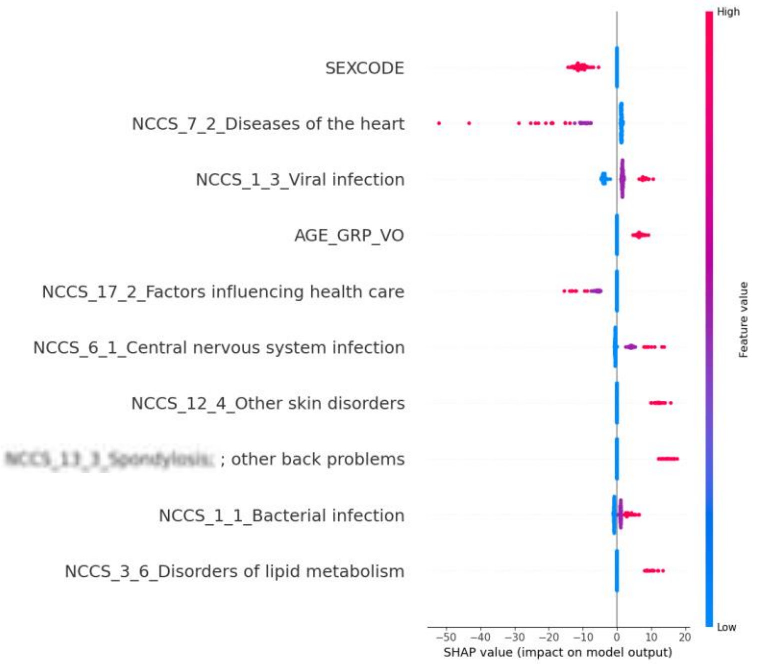

Figure S17. Time to ESRD predictions **a.**CKD1&2 to ESRD prediction results of 5 fold cross-validation for test data. Only clinical data was used for the cohort of 49,744 patients with CKD. The number of patients having the top 3 features is indicated as well as the uncensored patients. Concordance Index (CI) and Mean Average Error (MAE) of prediction results are shown together with the average number of days to ESRD.**b.** SHAP results with CCS Level 3 counts & Therapeutic group counts on CKD1&2. to ESRED predictions

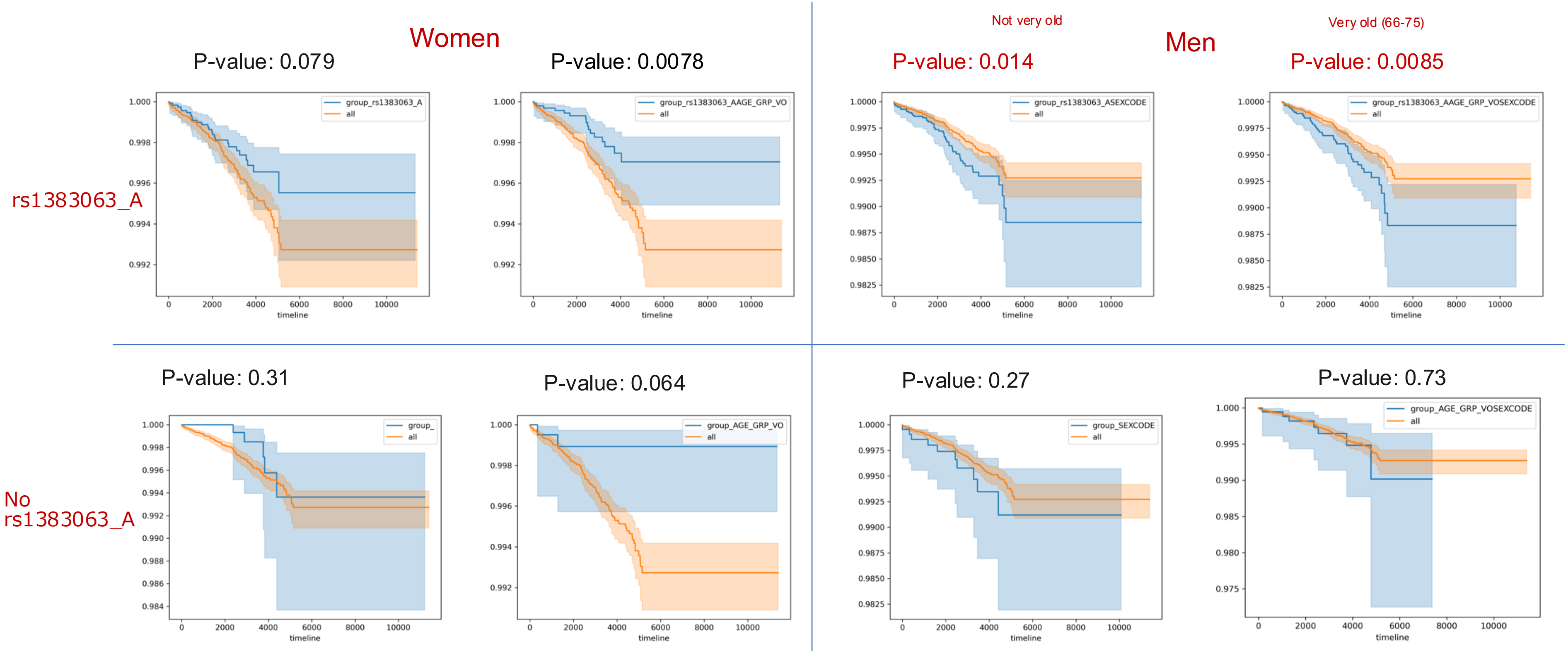

Figure S18. Kaplan Meier (KM) curves for survival predictions from CKD stage 1 & 2 to ESRD predictions using clinical and genomic data. Each KM curve represents parent populations in orange and subpopulations where the indicated feature is present (Sex, rs1383063_A and older age). Significant P-values are shown in red. Men with rs1383063_A have high risk of ESRD. Then, older (66-75) men with rs1383063_A have a higher risk.

	rs1383063_A	AGE_GRP_VO (66-75)	SEXCODE (Male:1, Female:0)	# of patients		# of uncensored patients		test_c_index		test_t_MAE		T2E	
				mean	std	mean	std	mean	std	mean	std	mean	std
Whole test dataset	-	-	-	9954.8	0.447	31.2	0.447	0.593	0.057	50.482	4.194	94.739	0.181
	0	0	0	504	22.627	1	0.707	0.744	0.062	36.320	10.810	94.525	2.375
	0	1	0	433.6	33.813	0.4	0.548	0.927	0.041	35.689	46.087	95.572	3.956
	0	1	1	379.8	17.196	1.4	1.140	0.702	0.201	34.299	27.946	96.902	2.103
	0	0	1	439	23.791	2	1.581	0.739	0.111	50.162	34.892	95.931	3.529
	1	0	0	2479.4	69.684	5.2	1.924	0.657	0.126	54.833	9.493	92.473	0.467
	1	1	0	2006.6	31.911	3.2	2.168	0.636	0.082	38.209	21.162	95.411	0.809
	1	1	1	1701	28.557	8.6	2.191	0.603	0.076	55.362	11.642	96.970	0.932
	1	0	1	2011.4	17.757	9.4	2.702	0.564	0.067	49.086	10.302	94.131	1.381

Figure S19. Feature information and prediction results for clinical and genomic data predictions of CKD 1&2 to ESRD time to event prediction. CKD12 results of 5cv for test data, the number of patients having the top 3 features is indicated as well as the uncensored patients. Concordance Index (CI) and Mean Average Error (MAE) of prediction results are shown together with the average number of days to ESRD.

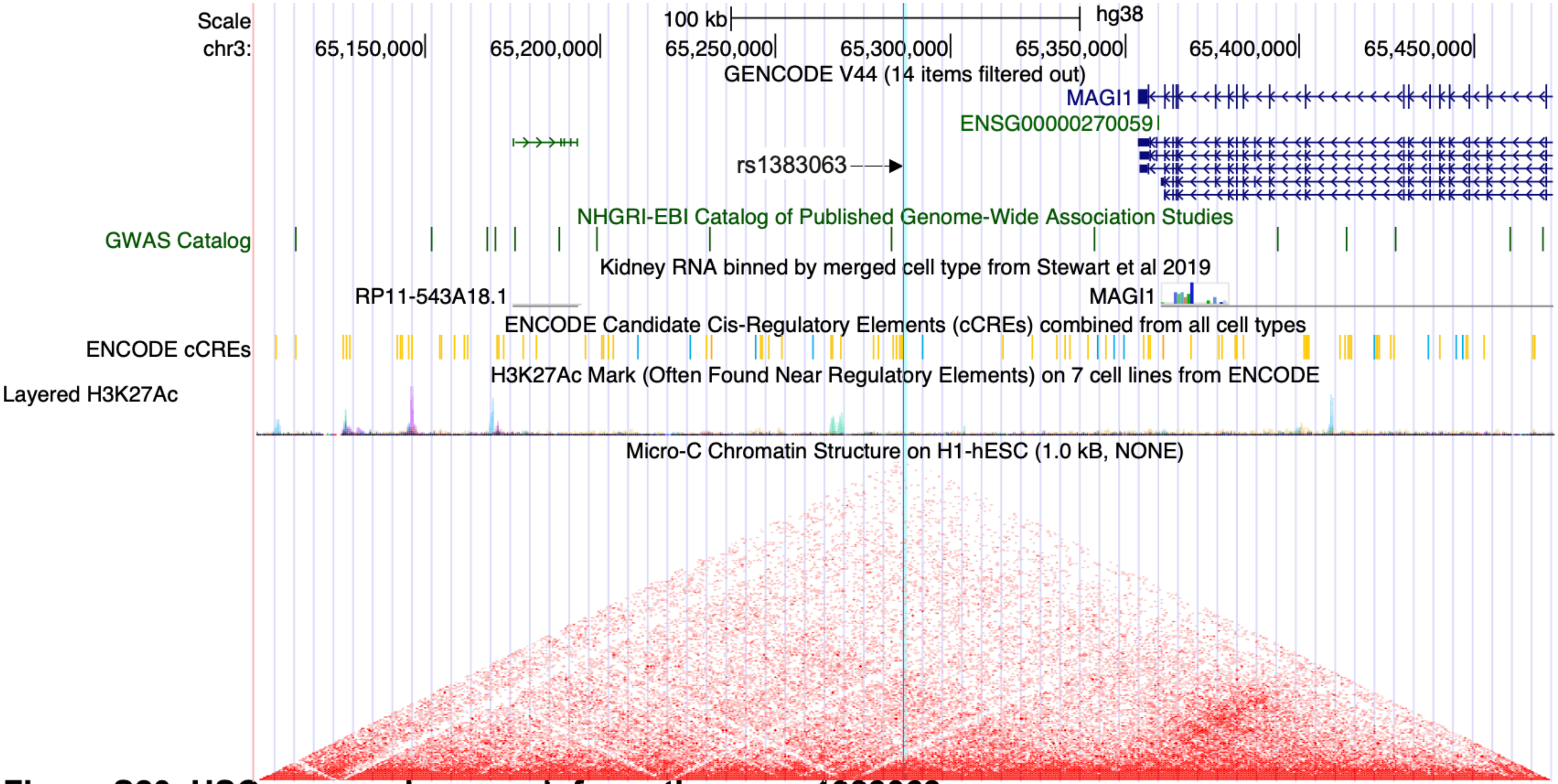

Figure S20. USC genome browser information on rs1383063.
MAGI1 gene position is indicated in dark blue, rs1383063 position is indicated by an arrow and vertical light blue line, GWAS catalog SNPs are shown in green, putative enhancers from ENCODE are shown in yellow, red triangle density indicates Topological Associated Domains as measured by microC. Scale is indicated above, as well as chromosomal coordinates.
